# Molecular Dynamics Simulation Study on the Cooling Behavior and Mechanical Properties of Silicone Carbide/Aluminum Composites

**DOI:** 10.3390/ma18163908

**Published:** 2025-08-21

**Authors:** Guanzhuo Zhou, Shiming Hao, Jingpei Xie, Hai Huang, Guopeng Zhang, Bin Cai, Yunjia Shi, Jing Wang, Jiefang Wang

**Affiliations:** 1 School of Physics, Zhengzhou University, Zhengzhou 450001, China; 2 School of Physics and Engineering, Henan University of Science and Technology, Luoyang 471023, China; 3 School of Materials Science and Engineering, Henan University of Science and Technology, Luoyang 471023, China

**Keywords:** cooling, mechanical properties, dislocations, molecular dynamics

## Abstract

The mismatch of the coefficient of thermal expansion (CTE) between the reinforcement and the matrix leads to thermal residual stresses and defects upon cooling from the processing temperature to room temperature. The residual stresses and defects have a significant impact on the mechanical properties of metal-matrix composites. To investigate the effect of cooling temperature on the residual stresses’ distribution and mechanical properties of SiC/Al, we investigated the cooling process of SiC/Al from different initial temperatures to room temperature. We found that residual stresses mainly distributed in the interface of SiC/Al composites after cooling, and the higher the initial temperature of cooling, the higher the value of residual stresses and the greater the degree of atomic displacement. During the cooling process, the Shockley partials and stair-rod dislocations were the two dominant dislocation structures. After cooling, the length of Shockley partials was about 80% and the length of stair-rod dislocations was about 18%. The mechanical properties of SiC/Al composites reduced after cooling. These results have filled the gap in understanding the mechanism of defect evolution in SiC/Al composites under cooling conditions, as well as the influence of cooling conditions on the mechanical properties of the material.

## 1. Introduction

Silicon carbide reinforced aluminum composites (SiC/Al) are extensively employed in aerospace, automotive, and electronic packaging applications owing to their advantageous properties, such as lightweight, high specific strength, and excellent corrosion resistance. During cooling from high manufacturing (e.g., 973 K) or annealing temperatures (e.g., 823 K) to room temperature (293 K), significant residual stresses develop in SiC/Al due to the mismatch of the CTE between the aluminum matrix and SiC reinforcement [1,2,3], with particularly effect in the aluminum matrix. Once the residual stress exceeds the yield stress of the metal phase, stress mismatch, microcracks, and interface fracture may occur. These residual stresses critically influence both the processing behavior and mechanical performance of SiC/Al, potentially compromising their practical applications [4,5,6,7]. Consequently, investigating the evolution mechanism of SiC/Al during cooling is essential for facilitating their broader industrial implementation.

Numerical simulations at the atomic scale have proven effective for studying residual stresses [8,9,10]. Through finite element analysis, Bouafia et al. [11] demonstrated that residual stresses in SiC/Al composites were concentrated near the matrix–particle interface, while regions of the matrix farther from the interface remained largely unaffected. These stresses developed during cooling. Consequently, analyzing the evolution of microscopic residual stresses distribution and dislocation density during cooling provides a viable approach for deformation mechanisms in composite materials.

The generation of microscopic residual stresses in SiC/Al composites during manufacturing and service has been investigated, with significant implication for their mechanical, fatigue, creep, and processing properties. Through molecular dynamics simulation of nanoindentation, Liu et al. [12] demonstrated that residual stresses on the SiC side after relaxation ranged between 50 and 60 GPa. Their results revealed that while aluminum accommodated these stresses through plastic deformation, silicon carbide primarily relieved them via fracture mechanisms. Tensile stresses are generated in the matrix, while compressive stresses develop in the reinforcement. Lu et al. [13] fabricated fiber-reinforced SiC/Al composites via hot-press sintering and characterized the residual stresses contribution to tensile behavior. Their findings indicated a pronounced influence of residual stresses at low tensile loads, with diminishing effects as stress increased. Through atomistic simulations of idealized Cu/SiC composites, Xiong et al. [14] characterized the formation of residual stresses, dislocations, and incomplete stacking fault tetrahedra during cooling. Their results revealed an explosive generation of dislocations during cooling, establishing a nonlinear relationship between dislocation density and temperature. The study further identified Shockley partials and stair-rod dislocations as the predominant dislocation types in Cu/SiC; the hardening mechanism in Cu/SiC was mainly plastic deformation. However, depending on the type of aluminum in the matrix, it may undergo precipitation hardening and/or plastic deformation. In related work, Dandekar and Shin [15] employed combined molecular dynamics and finite element simulations to parameterize the tension-shear behavior of Al-SiC systems under high-temperature tensile and shear loading conditions. Despite extensive experimental and computational investigations of residual stresses in SiC/Al composites, the fundamental mechanisms of residual stresses distributions and dislocation evolution remain insufficiently explored.

While significant advancements have been made in residual stresses measurement techniques, accurately characterizing detailed stresses properties and thermally induced defects in composites remain experimentally challenging. Molecular dynamics simulations offer a powerful alternative for atomic-scale analysis of mechanical properties and defect evolution mechanisms. The influence of residual stresses and thermally induced defects on mechanical behavior require further elucidation. Hence, in this study, molecular dynamics simulations were used to examine residual stresses distribution, dislocations evolution during cooling, and their combined effects on mechanical properties in SiC/Al composites. The findings provide fundamental insights for optimizing the performance of SiC/Al composite systems.

## 2. Computational Models and Methods

### 2.1. Simulation Details

**Figure 1:**
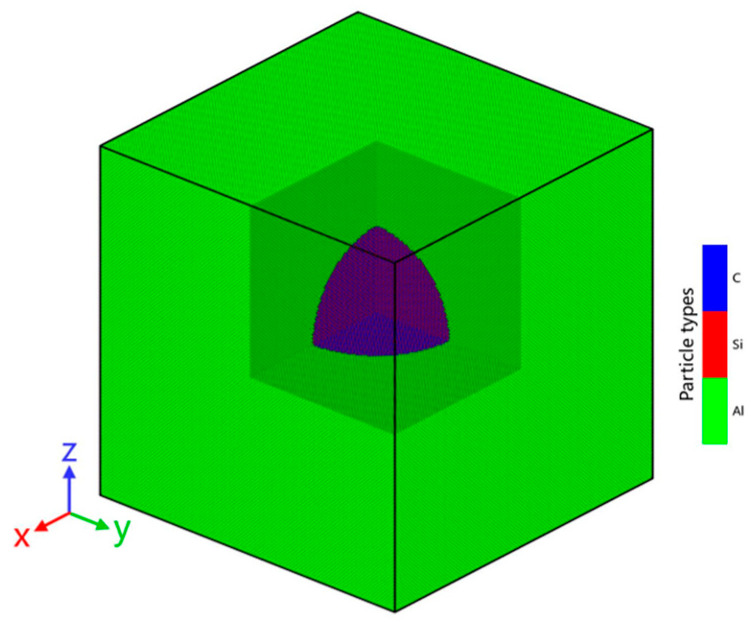
Cooling model of SiC/Al: Al atoms colored green, C atoms colored blue, Si atoms colored red.

The molecular dynamics simulations were performed by using the open-source LAMMPS software (LAMMPS-64bit-2Aug2023-MSMPI.exe) [16]. The aluminum matrix used in this study is pure aluminum, replacing pure aluminum with aluminum alloy would make the simulation more accurate, but due to current limitations in computer simulation technology, this is not yet feasible. As illustrated in Figure 1, the simulation model consists of a 40.0 × 40.0 × 40.0 nm^3^ cubic aluminum matrix with a 20.0 nm diameter spherical SiC particle located at its geometric center. The x, y, and z coordinate system represents the lattice directions [100], [010], and [001], respectively. Although spherical SiC reduced stress concentration effects, interface bonding area, and load-transfer efficiency, the system comprises approximately 3.9 million atoms, ensuring sufficient size to minimize boundary effects. During the molecular dynamics simulation process, the temperature is measured in Kelvins. Four thermal models were constructed with cooling from initial temperatures of 900 K, 800 K, 700 K, and 600 K to 300 K, along with an uncooled reference model at 300 K. To enhance physical accuracy, temperature-dependent lattice constants were calculated using the EAM potential for aluminum [17] and the Vashishta potential for SiC [18], with detailed results presented in Table 1.

Following model construction, energy minimization was performed using the conjugate gradient algorithm. The SiC/Al system underwent complete relaxation in an isothermal–isobaric (NPT) ensemble at target temperatures under zero external pressure, with a 120 ps equilibration period to achieve steady-state conditions prior to cooling. All models except the reference model were cooled uniformly from their respective initial temperatures (600 K, 700 K, 800 K, or 900 K) to 300 K at a constant rate of 10^12^ K/s. Each system underwent an additional 120 ps relaxation phase to ensure stability after cooling, with simulations conducted using a fixed 1 fs timestep. Tensile testing was performed in the NPT ensemble by applying uniaxial deformation along the *z*-axis at an engineering strain rate of 0.002 ps-1. The whole process was completed with the Nose–Hoover thermostat to ensure temperature stability. Residual stresses were quantified through equivalent stress analysis, combining normal and shear stress components, with Von Mises stress calculated as follows [19]:

(1) $$\sigma_{\mathrm{von}}^{2}=3\left(\sigma_{xy}^{2}+\sigma_{xz}^{2}+\sigma_{yz}^{2}\right)+\frac{1}{2}\left[{\left(\sigma_{xx}-\sigma_{yy}\right)}^{2}+{\left(\sigma_{xx}-\sigma_{zz}\right)}^{2}+{\left(\sigma_{yy}-\sigma_{zz}\right)}^{2}\right]$$

where $\sigma_{ij}$ represents the virial stress tensor component for individual atoms within the model. To reduce statistical fluctuations, the residual stress of atom *i* is calculated as follows [20]:

(2) $$\sigma^{avg}\left(i\right)=\frac{1}{N}\sum_{j=1}^{N}\sigma \left(j\right)$$

where $\sigma \left(j\right)$ represents the averaged virial stress tensor components for all atoms located within a 10 Å truncation radius centered on atom *i*, corresponding to the local virial stress distribution within this spherical domain.

The open-source visualization software OVITO (Version 3.11.3) [21,22] was employed to analyze atomic structure evolution. The Dislocation Extraction Algorithm (DXA) [23] was implemented to characterize dislocation networks and interfacial structural evolution, successfully identifying both primary dislocations and secondary grain boundary dislocations [24].

### 2.2. Interatomic Potentials

Molecular dynamics simulations enable complete determination of atomic structure evolution based on initial atomic positions and velocities, necessitating precise parameterization for reliable results. Three distinct interatomic potential functions were employed in this study: (1) The Embedded Atom Method (EAM) potential developed by Mishin et al. [17] for Al–Al interactions; (2) The Vashishta potential [18] for Si-C interactions; and (3) Morse potentials [25] for both Si-Al and C-Al interactions. The mathematical formulation of the Morse potential energy (E) is presented below:

(3) $$E=D_{0}\left[e^{-2\alpha \left(r-r_{0}\right)}-2e^{-\alpha \left(r-r_{0}\right)}\right]$$

where E denotes the potential energy, $D_{0}$ represents the potential well depth, α characterizes the potential well width, r indicates the interatomic distance, and $r_{0}$ corresponds to the equilibrium bond distance. All relevant parameters are systematically tabulated in Table 2.

## 3. Results and Discussion

### 3.1. Residual Stresses Distribution

**Figure 2:**
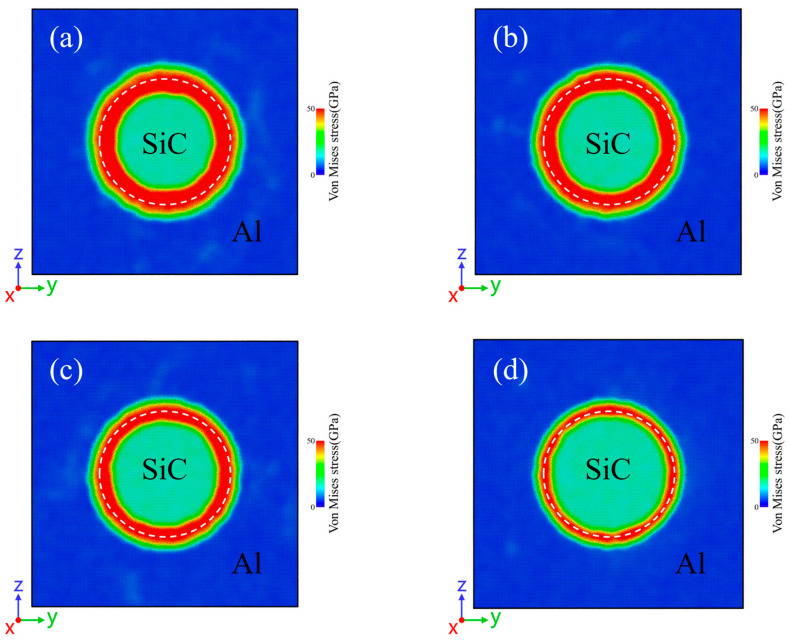
Cross-section of residual stresses distribution in SiC/Al cooled from different temperatures to 300 K: (**a**) 900 K, (**b**) 800 K, (**c**) 700 K, (**d**) 300 K.

During fabrication and service, SiC/Al composites undergo cooling from high temperatures to room temperature, generating residual stresses due to the significant mismatch of the CTE between the Al matrix and SiC reinforcement [26,27]. Figure 2 presents the Von Mises stress distributions in SiC/Al composites after cooling from various initial temperatures, where white dashed lines delineate SiC particle boundaries and color gradients indicate stress magnitude in the yz cross-sections. All interfaces between the Al matrix and SiC particles exhibit high residual stress concentrations. Notably, higher initial cooling temperatures result in greater residual stresses at the interface and a larger affected area. The 900 K cooling case demonstrates the most pronounced effect, exhibiting the largest stress values and influence range among the four models, confirming that high initial temperatures amplify both the thermal mismatch effect and interfacial stress accumulation. Figure 2d displays the reference model with zero temperature change, which theoretically should be stress-free. However, significant residual stresses persist at the interface, primarily arising from Al-C and Al-Si atomic interactions rather than thermal effects. These intrinsic stresses stem from chemical bonding and lattice mismatch between different atoms. While interfacial residual stresses exist at the interface in the uncooled state, their magnitude is still much lower than that of the cooled model. These findings demonstrate that interfacial residual stresses originate from both thermal mismatch effects and inherent interfacial characteristics of heterogeneous material systems.

**Figure 3:**
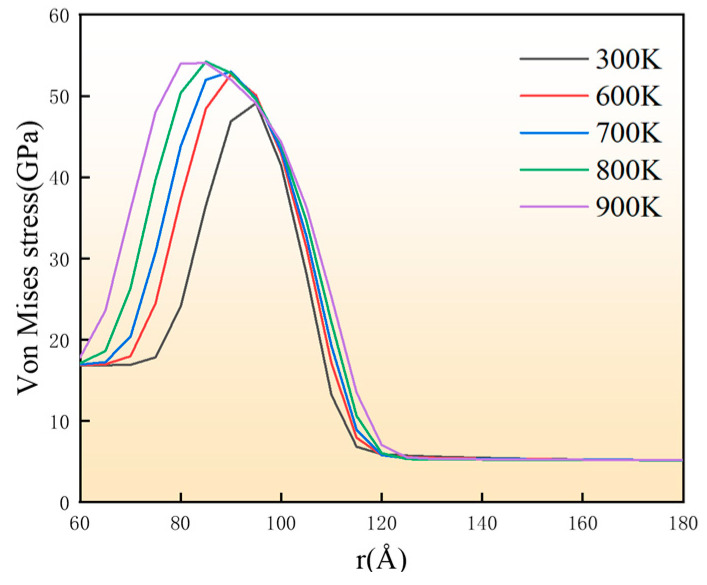
Radial distribution curves of residual stresses in SiC/Al after cooling at different temperatures.

To systematically analyze residual stresses distribution, spherical SiC particles were selected as coordinate origins for models cooled from different temperatures. Each particle was radially sectioned into 5 Å-thick shells, with average residual stresses calculated for each shell (see Figure 3). Interfacial regions exhibit residual stresses reaching 50 GPa, consistent with simulations by Liu et al. [12] but substantially exceeding experimental measurements [28]. This difference likely arises from the idealized conditions in molecular dynamics simulations, including reduced defects density, limited model size, and short simulation time. The 300 K model served as a benchmark to effectively standardize residual stresses in other models. The stress analysis showed a peak at the interface (~100 Å in Figure 3), which rapidly reduced with distance, corroborating experimental observations of interfacial stress concentration [29,30].

**Figure 4:**
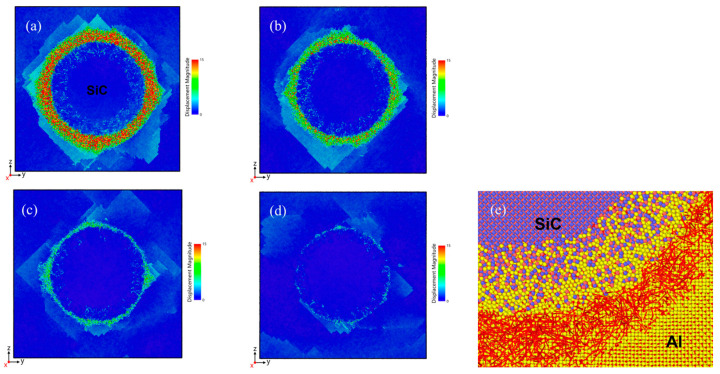
Variations in atomic displacement from different temperatures cooling to 300 K: (**a**) 900 K, (**b**) 800 K, (**c**) 700 K, (**d**) 600 K, (**e**) Red arrows indicate each atom’s displacement from its position after cooling from 900 K, with arrow length proportional to displacement magnitude.

Figure 4 presents the atomic displacement variations of SiC and Al atoms, with Figure 4e showing a magnified view of the region marked by the white frame in Figure 4a. The analysis reveals non-uniform thermal contraction, where interfacial atoms exhibit greater displacement magnitudes that scale with initial temperature. Al atoms move in all directions relative to SiC due to high initial temperature and interface diffusion during cooling. This thermally activated diffusion process shows strong temperature dependence, becoming negligible at lower temperatures. Cross-section analysis indicates a “square” displacement profile, characteristic of the {111}<110> slip system in face-centered cubic (fcc) metals. The compositional mismatch induces dislocation formation and stacking faults in the Al matrix. Notably, Figure 4e demonstrates significantly larger and more disordered Al atom displacements at the Al-SiC interface, resulting from combined thermal and interfacial effects that drive atomic diffusion into the reinforcement phase. The presence of incoherent precipitates likely increases the density of discontinuities in the composite, primarily through two mechanisms: the lattice mismatch at incoherent interfaces generates localized stress fields, promoting dislocation nucleation and pile-ups (Orowan mechanism), thereby raising dislocation density; weak bonding at incoherent interfaces micropore formation under mechanical/thermal loading.

### 3.2. Evolution of Dislocation Density During Cooling

**Figure 5:**
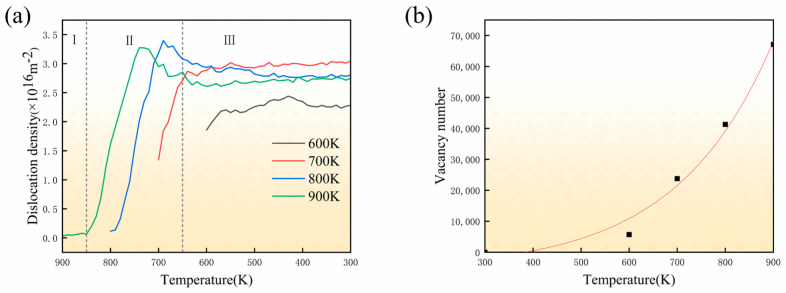
(**a**) Evolution of dislocation density with temperature; (**b**) Vacancy number at different temperatures after relaxation. I: initial stage, II: rapid-increase stage, III: stabilization.

Figure 5a illustrates the evolution of dislocation density in SiC/Al composites during cooling from various initial temperatures to 300 K, where dislocation density is quantified as the total dislocation line length per unit volume. Notably, dislocations form exclusively in the Al matrix while remaining absent in SiC reinforcement. The temperature-dependent evolution exhibits three characteristic stages: (1) an initial slow-growth stage with suppressed nucleation, (2) a subsequent rapid-increase stage, and (3) a final stabilization stage. For the case of cooling from 900 K, the dislocation density exhibits minimal change during the first stage (900–850 K). This is because the temperature is close to Al’s melting point (933 K), where intense atomic thermal motion disrupts the matrix structure and suppresses dislocation formation. Additionally, high-temperature dislocation annihilation further limits the accumulation of dislocations. In the second stage (850–700 K), the number of dislocations increased rapidly, and the dislocation density rose from 0.064 × 10^16^ to 3.273 × 10^16^ m^−2^. In the third stage, the dislocation density tends to stabilize, stabilizing at around 2.749 × 10^16^ m^−2^ at 300 K.

The rapid increase in dislocation density observed in the second stage may be attributed to the large number of vacancies during cooling from temperature close to the melting point. These vacancies aggregate into clusters that facilitate dislocation ring nucleation, as quantified by Wigner–Seitz analysis in Figure 5b. Residual stresses from thermal expansion mismatch between components further promote dislocation nucleation. Elevated temperatures also reduce lattice resistance and dislocation motion energy barriers through intensified lattice vibrations, collectively accelerating dislocation nucleation and multiplication. During the third stage, models cooled from high temperatures exhibit stable dislocation density, while the model cooled from 600 K shows moderate but consistently lower density increases, indicating the metal matrix’s initial thermomechanical state critically influences defect evolution. The calculated dislocation density (~10^16^ m^−2^) exceeds experimental values, likely due to scale limitations in molecular dynamics simulations. The dislocations present in the initial stage at 600–700 K may be due to the interaction between Al and SiC.

### 3.3. Dislocation Type

**Figure 6:**
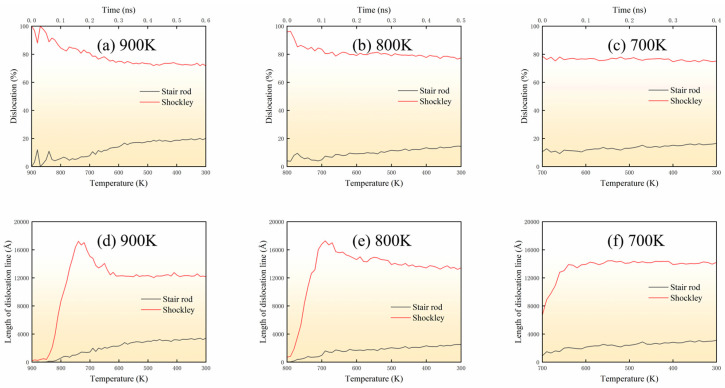
Proportion of Shockley partials and stair-rod dislocations with temperature and dislocation line length as a function of temperature: (**a**,**d**) 900 K→300 K, (**b**,**e**) 800 K→300 K, (**c**,**f**) 700 K→300 K.

Figure 6 presents the temperature-dependent evolution of two characteristic dislocation types in Al: 1/6<112> Shockley partials and 1/6<110> stair-rod dislocations. Dislocation proportion was quantified as the ratio of each type’s length to the total dislocation line length in the matrix. Figure 6a–c display the dislocation proportion evolution, while Figure 6d–f show dislocation line length variations. During cooling from 900 K, Shockley partials (≈80%) nucleate first due to perfect dislocation dissociation in the fcc lattice, followed by gradual stair-rod dislocation development (≈18%) through cross-slip and reactions. The remaining ≈2% comprises negligible minor types. Shockley partial lengths peak at ≈17,000 Å near 700 K before stabilizing. Below 700 K, both proportion and lengths stabilize as thermal activation diminishes, with length stabilization attributed to suppressed dislocation motion at lower temperatures. The persistent Shockley partial dominance reflects Al’s low stacking fault energy, while stair-rod formation indicates increasing dislocation network complexity.

**Figure 7:**
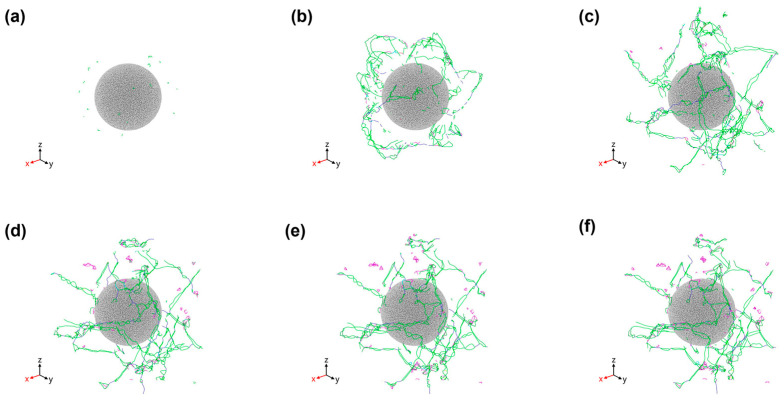
Evolution of defects during cooling at 900 K. The center spheres are SiC particles and the Al matrix has been removed: (**a**) 900 K, (**b**) 800 K, (**c**) 700 K, (**d**) 600 K, (**e**) 500 K, (**f**) 300 K.

Figure 7 illustrates the evolution of dislocation structures in SiC/Al composites during cooling from 900 K to 300 K, as analyzed through DXA technique in OVITO (Shockley partials: green; stair-rod dislocations: purple). Initial observations at 900 K reveal Shockley partials nucleating near SiC particles, attributed to interfacial Al-SiC interactions during relaxation. The combined effects of CTE mismatch and lattice mismatch generate localized stress fields that facilitate Shockley partial pre-nucleation. Progressive cooling induces substantial Shockley partial multiplication and limited stair-rod dislocations formation near reinforcement particles, demonstrating thermal mismatch stress accumulation as a primary driver for dislocation nucleation and propagation. By 700 K, dislocations propagate into the Al matrix boundary regions. Both dislocation types exhibit continuous temperature-dependent development, with quantitative evolution patterns (see Figure 6) confirming the observed structural changes. The persistent Shockley partial dominance (≈80% population) reflects Al’s low stacking fault energy, while stair-rod formation (≈18%) indicates increasing dislocation network complexity through cross-slip and reaction mechanisms.

### 3.4. Effect of Cooling on Tensile Properties

**Figure 8:**
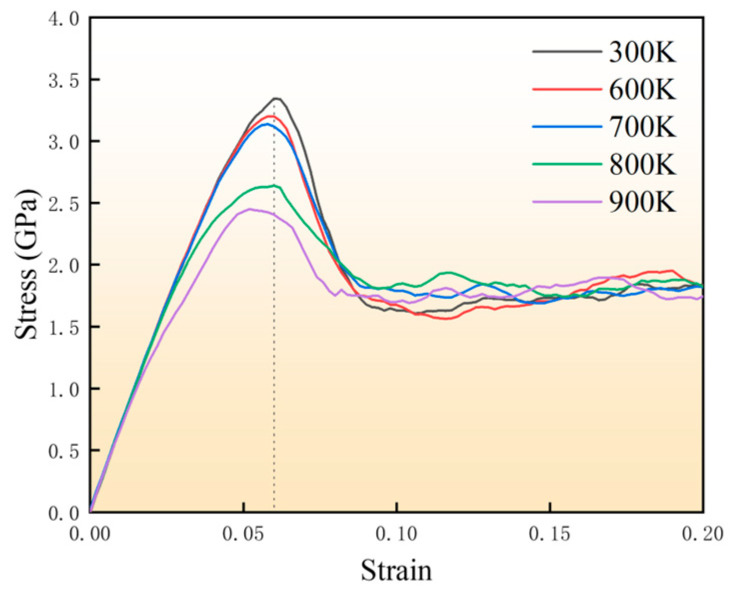
Uniaxial tensile stress–strain curves of SiC/Al composites after cooling at different initial temperatures.

Figure 8 presents the stress–strain behavior of SiC/Al composites cooled from various initial temperatures under uniaxial tensile loading, with an uncooled 300 K model serving as the control. The elastic deformation phase reveals decreasing stress–strain curve slopes with higher cooling temperatures, demonstrating more significant stress reduction in specimens cooled from elevated temperatures due to greater residual stresses and defect densities. Yield stress, defined as the peak stress, decreases progressively from 3.34 GPa (300 K model) to 2.45 GPa (900 K model). Cooling also induces earlier yielding, with yield strains reducing from ε = 0.06 (i.e., 300 K) to ε = 0.052 (i.e., 900 K). These results demonstrate that residual stresses and thermally generated defects collectively diminish both yield strain and strength. The superposition of residual and applied stresses creates localized stress concentrations that accelerate yielding and plastic deformation. Furthermore, thermal defects facilitate dislocation motion and crack nucleation, thereby compromising the material’s mechanical performance.

**Figure 9:**
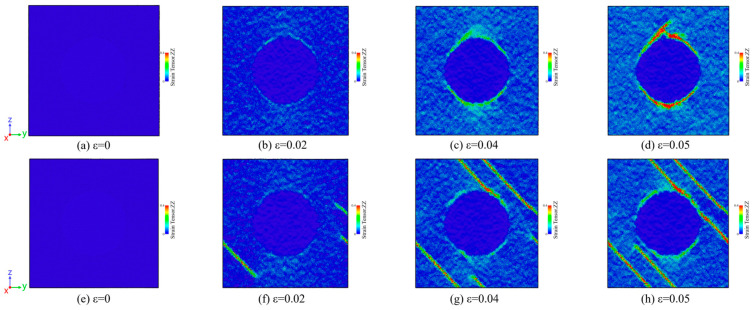
Snapshots of atomic cross-section strain tensor of SiC/Al composites at 300 K model at strains (**a**) ε = 0, (**b**) ε = 0.02, (**c**) ε = 0.04, (**d**) ε = 0.05 and 900 K model at strains of (**e**) ε = 0, (**f**) ε = 0.02, (**g**) ε = 0.04, (**h**) ε = 0.05.

Figure 9 presents strain tensor snapshots of atomic cross-sections for both 300 K model and 900 K model under uniaxial tensile loading. At zero strain (ε = 0), both models exhibit uniform dark blue coloration, indicating no observable local deformation. Initial deformation emerges at ε = 0.02, predominantly concentrated at SiC/Al interfaces, with the 900 K model demonstrating significantly greater deformation magnitude. At ε = 0.04, the 300 K model shows tensile-direction expansion, while the 900 K model exhibits more extensive strain distribution along face-centered cubic slip systems, confirming that high-temperature cooling enhances dislocation slip activation and localized plasticity. By ε = 0.05, interfacial deformation intensifies in the 300 K model, whereas the 900 K model displays dislocation propagation to edge regions, demonstrating cooling-induced dislocation extension and plastic deformation globalization. Throughout tensile loading, SiC reinforcement particles maintain minimal deformation, functioning primarily as load-transfer agents while plastic deformation remains confined to the Al matrix.

## 4. Conclusions

In this paper, the model assumes homogeneous material properties and does not account for the following microstructural factors: the role of grain boundaries in stress concentration and dislocation pile-up may alter the distribution of residual stresses; potential phase transformations (e.g., precipitation hardening) or interface reactions (e.g., Al_4_C_3_ formation) in the aluminum matrix may influence thermal mismatch stresses; common pores and defects in the processing process may act as stress con-centration sources or dislocation sources, thereby regulating plastic behavior and stress relaxation; and trace impurities in the aluminum matrix or SiC reinforcing phase may segregate at interface, altering diffusion kinetics and mechanical properties, while atomic diffusion at the SiC/Al interface at high temperatures may alleviate residual stresses through creep or interface slip mechanisms. These overlooked microstructural mechanisms collectively constrain the evolution of the actual stress state in composite materials. These simplifications may lead to deviations between simulation results and experimental results. Future work should combine phase field methods or multiscale modeling to address these effects, while conducting experimental verification (e.g., TEM for dislocation structures or XRD for residual stresses mapping).

In summary, SiC/Al composites is simplified to a model of a cubic metal matrix containing spherical reinforcement by molecular dynamics simulations to investigate the defect changes after cooling from different temperatures. From the simulation results, it is observed that the cooling process introduces residual stresses in SiC/Al composites, and the residual stresses are mainly concentrated in the interface region between the Al matrix and the SiC reinforcement. The higher the initial cooling temperature, the higher the value the residual stress near the interface; in addition, high-temperature cooling leads to more significant changes in atomic displacement near the interface, and the Al atoms at the interface move in all directions and undergo a greater degree of deformation, with the displacement profile approximating a “square”. A large number of dislocation defects are generated inside the material during high-temperature cooling. The evolution of the dislocation density can be divided into three stages: in the first stage, the dislocation density is almost zero; in the second stage, the dislocation density increases rapidly, and the dislocation nucleation and proliferation are significant; in the third stage, the dislocation density tends to stabilize. The thermodynamic state of the composite material plays a crucial role in the generation and evolution of defects, and at the same time, the change in dislocations is the result of the coupling of many factors. With the evolution of defects in Al/SiC composites during the cooling process, some interesting phenomena were found. During the cooling process, the types of dislocations are mainly dominated by 1/6<112> Shockley partials and 1/6<110> stair-rod dislocations, in which the Shockley partials are dominant with a percentage of about 80%. During the cooling process, the dislocations are first generated near the interface, and with the decrease in temperature, the dislocations are gradually extended to the boundary region of the Al matrix. In addition, by performing tensile simulations of the cooled model, the pre-existing residual stresses and thermogenic defects in the model cause the model yield strain to advance and the yield stress to decrease, and the stress reduction is more significant when cooling from higher temperatures.

## Figures and Tables

**Table 1:** Lattice constants at different temperatures.

Temperature (K)	300	600	700	800	900
Al (Å)	4.065	4.086	4.095	4.103	4.112
3C-SiC (Å)	4.371	4.383	4.389	4.393	4.396

**Table 2:** Functional parameters of the Morse potential by Zhao et al. [25].

Atom Pair		
Al-Si	*D*_0_ (eV)	0.4824
	*α* (1/Å)	1.322
	*r*_0_ (1/Å)	2.92
Al-C	*D*_0_ (eV)	0.4691
	*α* (1/Å)	1.738
	*r*_0_ (1/Å)	2.246

## Data Availability

The original contributions presented in this study are included in the article. Further inquiries can be directed to the corresponding author.
